# A mitochondrial location for haemoglobins—Dynamic distribution in ageing and Parkinson's disease^☆^

**DOI:** 10.1016/j.mito.2013.12.001

**Published:** 2014-01

**Authors:** Freya Shephard, Oliver Greville-Heygate, Oliver Marsh, Susan Anderson, Lisa Chakrabarti

**Affiliations:** a School of Veterinary Medicine and Science, Faculty of Medicine and Health Sciences, University of Nottingham, LE12 5RD, United Kingdom; b Graduate Entry Medicine, University of Nottingham, DE22 3DT, United Kingdom

**Keywords:** Ageing, Haemoglobin, Mitochondria, Neurodegeneration, Parkinson's Disease

## Abstract

Haemoglobins are iron-containing proteins that transport oxygen in the blood of most vertebrates. The mitochondrion is the cellular organelle which consumes oxygen in order to synthesise ATP. Mitochondrial dysfunction is implicated in neurodegeneration and ageing. We find that α and β haemoglobin (Hba and Hbb) proteins are altered in their distribution in mitochondrial fractions from degenerating brain. We demonstrate that both Hba and Hbb are co-localised with the mitochondrion in mammalian brain. The precise localisation of the Hbs is within the inner membrane space and associated with inner mitochondrial membrane. Relative mitochondrial to cytoplasmic ratios of Hba and Hbb show changing distributions of these proteins during the process of neurodegeneration in the *pcd^5j^* mouse brain. A significant difference in mitochondrial Hba and Hbb content in the mitochondrial fraction is seen at 31 days after birth, this corresponds to a stage when dynamic neuronal loss is measured to be greatest in the Purkinje Cell Degeneration mouse. We also report changes in mitochondrial Hba and Hbb levels in ageing brain and muscle. Significant differences in mitochondrial Hba and Hbb can be seen when comparing aged brain to muscle, suggesting tissue specific functions of these proteins in the mitochondrion. In muscle there are significant differences between Hba levels in old and young mitochondria. To understand whether the changes detected in mitochondrial Hbs are of clinical significance, we examined Parkinson's disease brain, immunohistochemistry studies suggest that cell bodies in the substantia nigra accumulate mitochondrial Hb. However, western blotting of mitochondrial fractions from PD and control brains indicates significantly less Hb in PD brain mitochondria. One explanation could be a specific loss of cells containing mitochondria loaded with Hb proteins. Our study opens the door to an examination of the role of Hb function, within the context of the mitochondrion—in health and disease.

## Highlights

• Both Hba and Hbb are co-localised with the mitochondrion in mammalian brain.
• Hbs are located in the inter membrane space and inner mitochondrial membrane.
• α and β haemoglobin protein distribution changes with age in brain mitochondria.
• Mitochondrial fractions from PD brains contain significantly less Hb than controls.

## 1 Introduction

Mitochondrial dysfunction is currently under scrutiny for its role in neurodegeneration. It is important first to understand the basic mechanisms that trigger neuronal demise. We have utilised a classic mouse model of neurodegeneration to try and answer questions about the changes that occur early, or pre-symptomatically, in neurons that are destined to undergo a degenerative process. The mouse model Purkinje Cell Degeneration mouse (*pcd*), shows signs of ataxia soon after weaning (Chakrabarti et al., 2006). *Pcd* is a natural model of ataxia, resulting from a rapid and complete loss of Purkinje cells in the cerebellum. From about 5 months of age, ataxia is accompanied by other neuronal losses, notably in the photoreceptors of the eye (Chakrabarti et al., 2008). The male homozygous mouse is infertile. We and others have confirmed that the *pcd* phenotype is due to the loss of a single protein Nna1/CCP1 which is predicted to have zinc-carboxypeptidase activity (Chakrabarti et al., 2008). Much of the research effort focused on the degeneration of neurons has relied upon the construction of mammalian models using transgenic, knockout and other manipulations to simulate the rarer, but more predictable, forms of inherited human neurodegenerative disease. We now have an arsenal of genetic factors that should predict a neurodegenerative outcome (Cookson, 2012). However, a significant drawback of these approaches has been the lack of genotype and phenotype correlation between man and mouse. It is recognised that naturally occurring instances of disease in model organisms can often present a closer approximation to spontaneous disease in humans (Barbaric et al., 2007). We have been able to use this correlation in our study of *pcd*, by linking the degeneration phenotype in this spontaneously occurring mouse model with specific mitochondrial dysfunction and autophagy (Chakrabarti et al., 2009, 2010). Our characterisation of a mitochondrial defect was echoed in the fruit fly ortholog NnaD, and therefore we demonstrate that important neurodegenerative disease pathways can be common across species and are likely to be relevant to a number of human diseases.

In the current study we build on our *pcd* proteome data and focus upon proteins in fractions enriched for mitochondria. Previously, we have shown how mitochondria are morphologically different and undergo mitophagy in Purkinje cells destined to degenerate (Chakrabarti et al., 2009). We have also shown, by biochemical methods, how the function of mitochondria extracted from pre-symptomatic tissues appears to be compromised, measured by the activity of complex I enzymes (Chakrabarti et al., 2010). These symptoms of mitochondrial dysfunction are also features of common neurodegenerative disease in man (Rugarli and Langer, 2012; Yan et al., 2012). The approach described herein was employed to elucidate the specific factors within, and closely associated with, mitochondrial organelles. These factors are important to understand the molecular basis of neurodegenerative disease. Haemoglobins were one group of proteins in our datasets examining mitochondria from neurons; novel candidates in the process of neurodegeneration. The distribution of these, otherwise well characterised, proteins was noticed as a significant difference in the mitochondria between control and degenerating tissues.

The data presented herein arise from an ongoing proteomics study to determine differentially expressed proteins isolated from neuronal tissue from a mouse model of neurodegeneration (*pcd^5j^*) (Chakrabarti et al., 2006). Two dimensional gel analyses of mitochondrial fractions from these tissues revealed a number of interesting spots which were analysed by mass spectrometry for protein identities. We were surprised to find both α and β haemoglobin (Hba and Hbb) proteins in these lists. We re-examined previously published proteomic datasets (generated from *pcd^5j^* whole retina samples) to determine retrospectively, whether changes in these proteins could also be found in data obtained by mass spectrometry based quantitative proteomics (Chakrabarti et al., 2010). Interestingly, Hb sub-units beta-1 and beta-2 were top hits in the biological process category ‘transport’—ranked first in weight and elimination. Hb subunit beta-1 was assigned a *pcd/wild type* ratio of 0.00389 (false discovery rate FDR 0.01) and Hb subunit beta-2 *pcd/wild* type ratio also showed a reduction (0.0041 FDR 0.01). Ratios for beta sub-units in retina show a marked overall reduction in *pcd^5j^* mitochondria, compared with controls. Mass spec ratios can be limited by individual protein ionisation efficiencies in global proteomics studies. We therefore decided to follow up on what could be an interesting and important finding using a variety of technical approaches.

Our discovery falls in well with the fact that haem is manufactured almost entirely within the mitochondrion, it is certainly reasonable that haem containing proteins may also reside in the organelle where they are constructed (Fleming and Hamza, 2011). Haem is synthesised via a series of pathways in the mitochondrion and cytoplasm. The primary and latter steps in the haem synthetic process are carried out inside the mitochondrion. The presence of oxygen binding proteins at the site of oxidative phosphorylation seems logical.

Haemoglobin has been located to within the neuron previously but not to any particular organelle (He et al., 2009; Schelshorn et al., 2009). The oxygen carrying protein was found to be present in neurons and not glial cells, in several regions of the brain. Interestingly, the levels of neuronal haemoglobin were upregulated by mimicking cerebral ischaemia both in vitro and in vivo. Other indications that haemoglobin has important intracellular roles include the antagonistic function of the haemoglobin derived neuropeptide Hemopressin, on the cannabinoid receptor CB1 (Gomes et al., 2009). CB1 has also recently been localised to the mitochondrion where they are postulated to regulate energy metabolism (Bénard et al., 2012).

Neuroglobin, a neuron specific oxygen carrier has been shown to associate in the mitochondrial fraction and to be located within mitochondria now we show that haemoglobins are embedded in those regions of the organelle where a continuous supply of oxygen is critical for survival (Lechauve et al., 2012; Yu et al., 2012). From an evolutionary point of view, for those that accept the endo-symbiotic theory of mitochondrial entry into an ancestral eukaryotic cell, an individual mitochondrion in most tissue types might do well to ensure a ‘reserve’ oxygen supply.

## 2 Results

### 2.1 HbA and HbB show intracellular co-localisation with mitochondria in human frontal lobe and substantia nigra

**Fig. 1:**
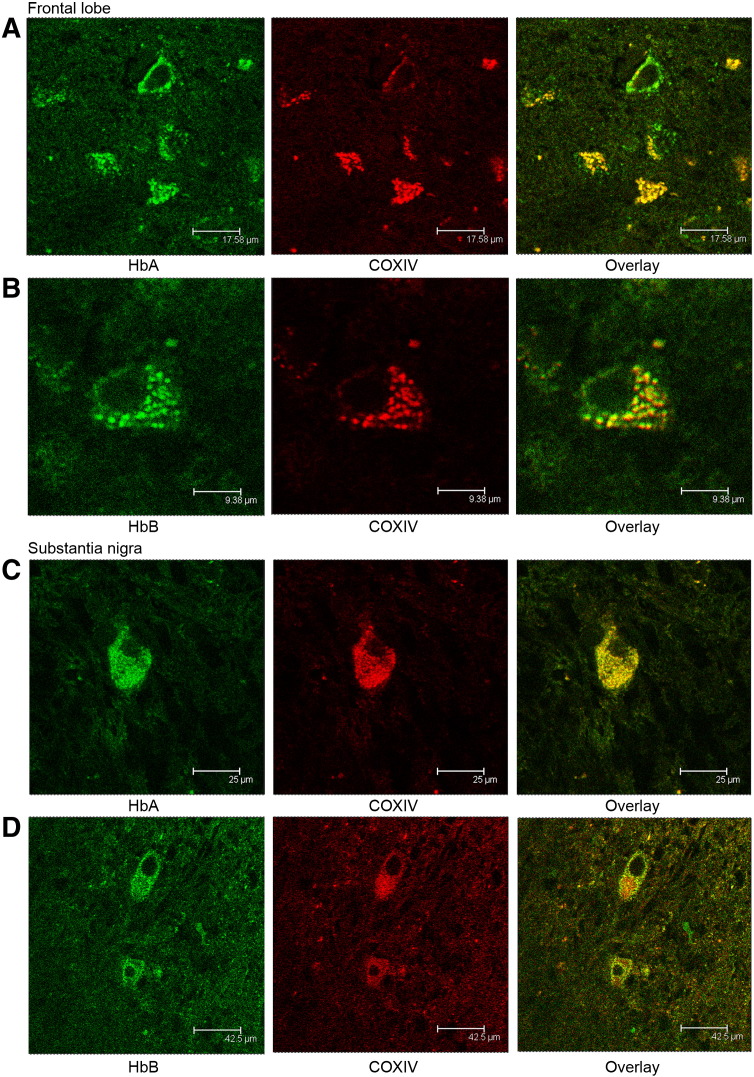
HbA and HbB colocalise with mitochondria in control human frontal lobe. Confocal images. Mitochondria are visualised with the COXIV antibody (red) and HbA and HbB (green) are overlaid to provide colocalisation information. Sections from human frontal lobe and substantia nigra were each investigated for both haemoglobins. A. HbA and COXIV colocalise in many regions of the frontal lobe, it appears that there are areas which are specific for HbA where mitochondria are not present. B. HbB and COXIV also overlap in expression in the frontal lobe, though again there are areas of HbB expression where mitochondria are not detected. C. In the substantia nigra HbA and COXIV expression also overlap, a close look suggests some mitochondria do not express Hba and there are areas of diffuse HbA expression that do not correspond to mitochondrial staining. D. Again in the substantia nigra there is considerable overlap of HbB and COXIV localisation, though there are distinct regions where either one or the other protein is expressed or colocalisation doesn't appear to be uniform.

In order to begin to understand whether the Hbs we detected in our global proteomics datasets and on our 2D gels were true positives or the result of erythrocyte contamination at the time of tissue collection, we used antibodies to both HbA and HbB to interrogate brain sections from non-degenerative human frontal lobe and substantia nigra. A second antibody marker was used in conjunction with HbA or HbB, COXIV—a mitochondrial marker (red colour). Frontal lobe sections from fixed human control brains were immunostained for HbA (Fig. 1A) and HbB (Fig. 1B). The central panel in each case shows the same sections double labelled with COXIV. The overlays indicate overlapping areas measuring ~ 1 μm which correlates well with the expected size of mitochondria—we think this is most readily estimated in the second set of panels (Fig. 1B). Again, in human control substantia nigra there is clear overlap of signal between COXIV and HbA (Fig. 1C) and HbB (Fig. 1D).

### 2.2 HbA is found within the inter-membrane space of the mitochondrion

**Fig. 2:**
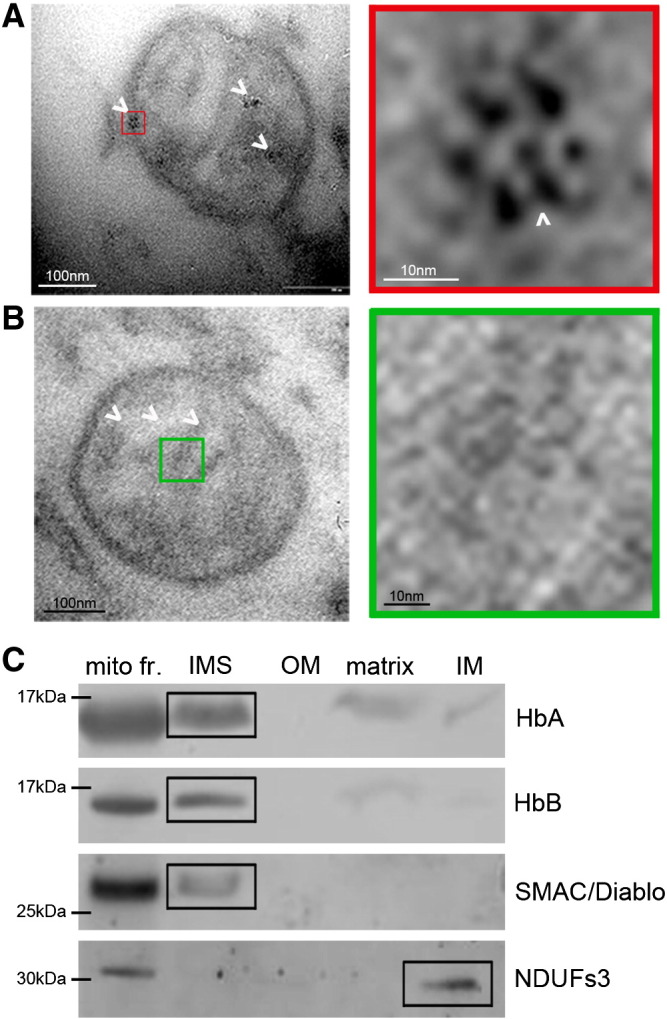
HbA and HbB are localised within the mitochondrion—to the inner membrane and inter-membrane space. A. HbA is localised (white arrows) to the mitochondrion and can be observed in the inter-membrane space and within cristae structures. A characteristic rosette pattern of gold particles is seen when immuno electron microscopy of HbA is performed. Immuno-electron microscopy was performed on control human brain tissues using 6 nm immunogold. B. Secondary antibody no primary antibody control-conjugated immunogold. C. HbA and HbB are co-isolated with inner membrane and inter-membrane space sub-fractions from human mitochondrial preparations. Smac/Diablo confirms isolation of inter-membrane space fractions. The inner membrane enrichment is confirmed by NDUFS3 reactivity, faint bands in this lane suggest that Hbs also associate with the mitochondrial inner membrane. Abbreviations used: mito fr.—whole mitochondrial fraction before sub-fractionation, IMS—inter-membrane space, OM—outer membrane, IM—inner membrane space.

Co-localisation of proteins with the mitochondrion is important to establish at a histological level but may only be indicative that the protein is associated with the outer surface of the organelle. There are few methods that allow unambiguous placement of a protein within an organelle such as the mitochondrion. We used immuno electron microscopy (immuno-EM) to localise HbA at an ultra-structural level in non-neurodegenerative (control), post-mortem human brain. We utilised a primary antibody directed against HbA, visualised with 6 nm gold particles—immunogold. HbA is visualised quite clearly (white arrows) within the inter-membrane space of the mitochondrion (Fig. 2A). A characteristic rosette arrangement of immunogold particles is apparent (see inset). A control experiment performed while omitting a primary antibody (Fig. 2B) demonstrates a lack of specific staining. Darker regions in mitochondria from control grids do not resolve into gold particles of the expected size. Adjacent regions with blood vessels, stained positive for HbA in erythrocytes, providing a positive control (data not shown).

A second method to determine the sub-mitochondrial localisation of proteins was used to confirm our findings from the immuno-EM. We subjected human brain enriched mitochondrial preparations to a sub-fractionation protocol. We confirm that fractions were enriched for the target sub-organellar locations, by the use of established antibody markers for those regions (Fig. 2C). Reactivity with both Hb antibodies appeared to lie primarily in the inter-membrane space with fainter bands corresponding to inner membrane and matrix fractions. This confirms the ultra-structural localisation data.

### 2.3 Hbs are significantly decreased in mitochondria in degenerating neurons

**Fig. 3:**
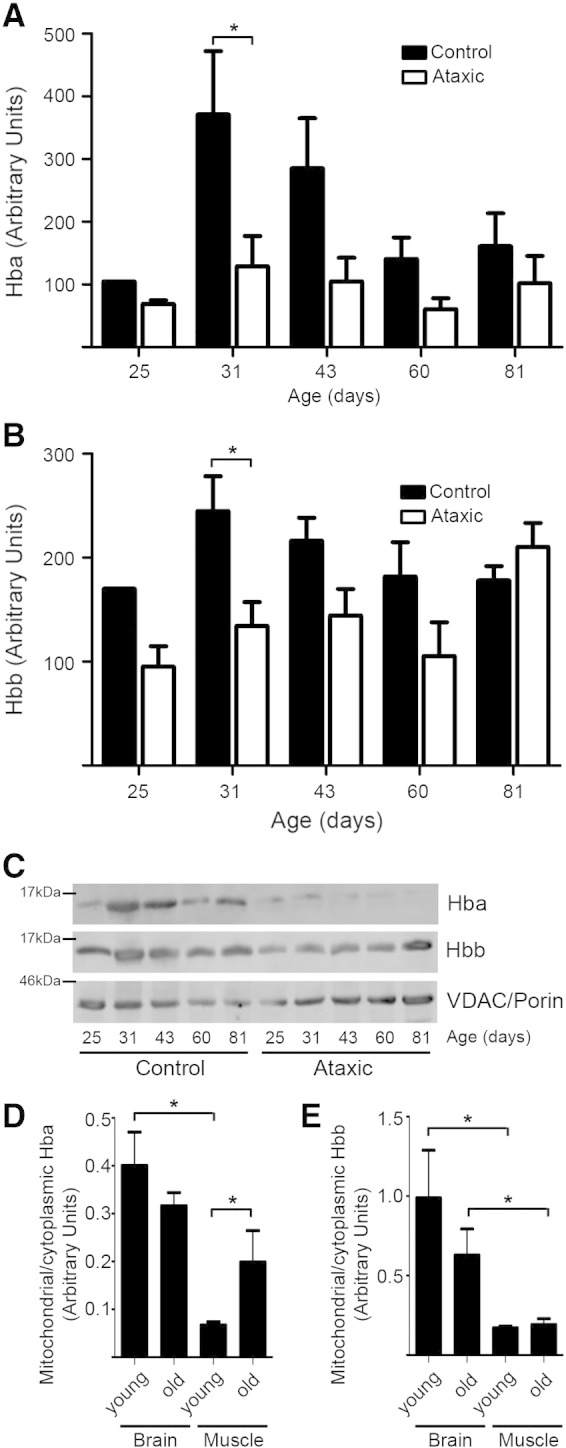
Comparison of mitochondrial Hba and Hbb in *pcd^5j^* during neurodegeneration and in normal, ageing mouse. A Hba and B Hbb relative quantities are shown in *pcd^5j^* (ataxic) and control mouse neuronal mitochondria at intervals from weaning to maturity. Both Hba and Hbb are reduced relatively in mitochondria in mouse brain undergoing a neurodegenerative process. The difference between ataxic and control samples reaches significance in the brains taken at 31 days. At this time point both Hba and Hbb are significantly reduced in ataxic mitochondria when compared with quantities in the cytoplasm. A + B *p < 0.05 (2-way anova) n = 3. C. Western blot example of Hbs variation through the process of degeneration. Each sample lane is first analysed, for normalisation, to determine total mitochondrial content ratio with respect to cytoplasmic contamination (as described for D and E below). Hbs are then compared with the normalised (compared to cytoplamic) VDAC/porin quantification in each case. D. Hba and E. Hbb were measured in mitochondrial and cytoplasmic fractions prepared from 8 week and 18 month old mouse brain and skeletal muscle. The ratio of Hba and Hbb in the mitochondrial fraction (normalised to porin) compared with the cytoplasm (normalised to hsp-90) is plotted. Significant differences between mitochondrial and cytoplasmic ratios of both Hbs were found between the two tissue types, brain and muscle. Hba shows a significant increase when measured in old muscle mitochondria compared with young muscle mitochondria. Data shown are mean ± SEM of 3 experiments *p < 0.05 (one-tailed *t*-test).

We measured the relative quantity of mitochondrial Hb in brain tissue from *pcd* and litter-mate control mice (data points were normalised against abundance of VDAC/Porin), starting at just after weaning (25 days) when the *pcd^5j^* homozygous mice begin to show an ataxic gait. Hb levels were quantified and are displayed in arbitrary units as a value normalised to the quantity of porin for that mitochondrial fraction. In control brain mitochondrial fractions Hba shows a trend toward up-regulation at around a month after birth. The *pcd^5j^* brain mitochondrial fractions do not reflect this trend and by 31 days after birth there is a significant difference between control mitochondrial Hba and Hba in the *pcd^5j^* brain mitochondria p < 0.05 (2-way ANOVA) n = 3 (Fig. 3A). Similarly at the 31 day time point Hbb in the *pcd^5j^* brain mitochondria is significantly less when compared with controls p < 0.05 (2-way ANOVA) n = 3 (Fig. 3B). By 81 days, when almost all Purkinje cells in the *pcd^5j^* cerebellum are lost, Hbb trends toward an increased level of expression in the mitochondrion but this does not reach significance. Results are expressed as the mean ± SEM of at least 3 independent experiments. A representative western blot is included (Fig. 3C) to demonstrate typical differences between the control and ataxic mitochondrial preparations.

### 2.4 Levels of Hba and Hbb protein expression in old and young mammalian skeletal muscle and brain suggest a complex interplay of Hb chain expression within the cell throughout the lifetime of an individual

We show that in mouse tissue samples obtained at 8 weeks of age (young) and 18 months (old) there appear to be differences in the ratio of mitochondrial versus cytoplasmic Hbs. The ratio of Hba and Hbb in the mitochondrial fraction (normalised to porin) compared with the cytoplasm (normalised to hsp-90) is plotted. Significant differences between mitochondrial and cytoplasmic ratios of both Hbs are seen when comparison is made between the two tissue types, brain and muscle. Hba shows a significant increase when measured in old muscle mitochondria compared with young muscle mitochondria. There are also significant differences seen in Hba content between young brain and young muscle mitochondria (Fig. 3D). Hbb levels across lifespan show no significant differences within the same tissue. However, brain mitochondrial Hbb content seems significantly higher than in muscle in both old and young tissues (Fig. 3E). Data shown are mean ± SEM of 3 experiments p < 0.05 (one-tailed *t*-test).

### 2.5 There is reduced mitochondrial Hb in Parkinson's disease (PD) mitochondria compared with Alzheimer's Disease (AD) or control (non-degenerative) brain mitochondria

**Fig. 4:**
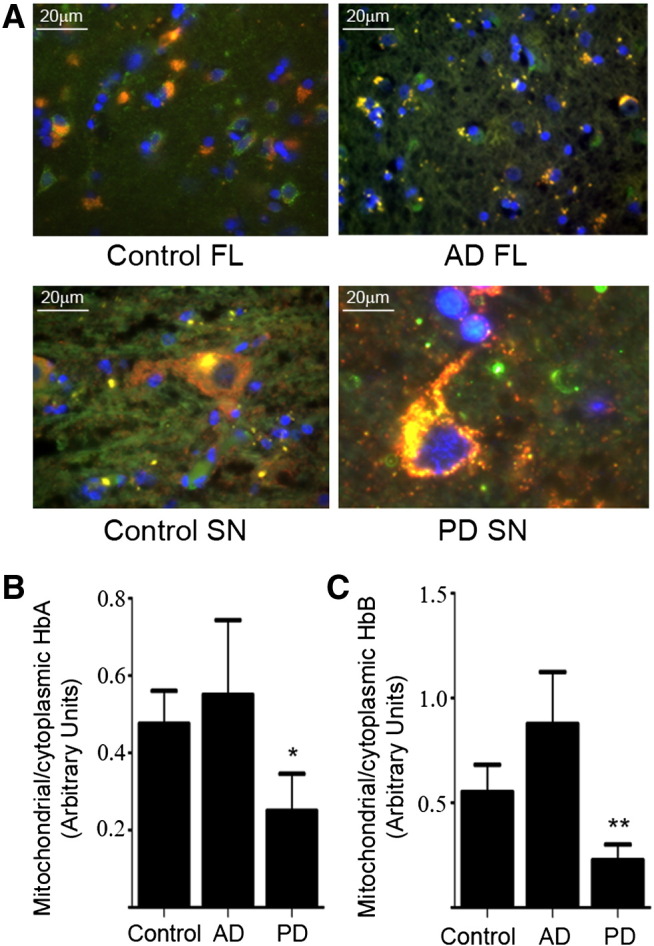
A. HbA colocalises with mitochondria in human brain regions frontal lobe and substantia nigra (HbA-green, COXIV-red, DAPI stain—blue). Control human frontal lobe is compared with Alzheimer's (AD) frontal lobe. There is a suggestion that in AD frontal lobe there is less diffuse mitochondrial staining than in controls with many more frequent clumps of mitochondrial and HbA colocalisation. In control substantia nigra large cell bodies show both separate staining and also colocalisation of HbA and COXIV. PD substantia nigra cell bodies also show overlap of these proteins, with perhaps a greater distribution of colocalisation both intra and extracellularly. B HbA mitochondrial to cytoplasmic ratio is reduced in PD brain *p = 0.065 (1 tail *t*-test) n = 4, compared with controls. There is no significant difference in AD mitochondrial/cytoplasmic ratios of HbA when compared with controls. C. HbB is significantly reduced in PD mitochondria compared with cytoplasmic HbB **p = 0.038 (1 tail *t*-test) n = 4. Again ratios in AD brain fractions do not show a significant change when compared with controls. Abbreviations: FL—frontal lobe, SN—subtantia nigra, AD—Alzheimer's Disease, PD—Parkinson's Disease.

Antibodies directed to both HbA (Fig. 4A) and HbB (not shown) demonstrate that mitochondria, identified with a COXIV antibody (red), colocalises with HbA (green, DAPI nuclear stain—blue). Frontal lobe of AD brain is shown with neuronal losses evident when compared with control frontal lobe tissue (Fig. 4A). Closer inspection reveals a clustering of doubly stained (yellow) intracellular components. In the AD frontal lobe sections it appears that some of the co-localised clusters are in extracellular areas. The substantia nigra of PD brains shows that the few remaining nigral neurons are doubly labelled throughout the cell body. Comparison with control substantia nigra suggests an increased quantity of HbA containing mitochondria in neurons that survive to late stages, or perhaps in those that are imminently due to degenerate.

We subjected mitochondrial and cytoplasmic fractions of control, AD and PD brain to western blotting to determine the relative quantity of Hb proteins within the mitochondria as a ratio; compared with the quantity in the cytosolic fraction (the nuclear fraction was not examined). We find that HbA mitochondrial to cytoplasmic ratio is reduced in PD brain p = 0.065 (1 tail *t*-test) n = 4. However, there is no significant difference in AD mitochondrial/cytoplasmic ratios of HbA, perhaps a suggestion of the opposite, that the ratio of HbA may increase (Fig. 4B). HbB is also found to be significantly reduced in PD mitochondria p = 0.038 (1 tail *t*-test) n = 4 (Fig. 4C). Again ratios in AD brain fractions do not show a significant change, but trend in the opposite direction to PD.

## 3 Discussion

We present data demonstrating the presence of Hba and Hbb proteins in the mitochondria of neurons and mammalian skeletal muscle. Our initial discovery resulted from a perceived difference in these proteins between degenerating brain mitochondria (*pcd^5j^*) revealed by proteomics analyses (Fig. 1). Our 2D proteomics data suggest that haemoglobin subunit beta-1 and haemoglobin subunit alpha are increased in mitochondria isolated from *pcd^5J^* brain at 27 days age. Later we show western blots indicating that at 31 days after birth (and indeed) through most of the time period measured Hba and Hbb seem to both be reduced in quantity in the brain mitochondria. The western blot data is most specific to the proteins interrogated. The mass spec identification of the beta-1 and beta-2 sub units in the 2D gels was also specific. This discrepancy in the relative expression seen using two different techniques could perhaps be a result of the other beta subunits (there are five in total) not being measured in the 2D gels yet still detected by the polyclonal antibody used in western blotting. The techniques also detect proteins using very different methods—antibodies versus mass spectrometry. Our intention here is to show that each technique does detect differences between degenerating and control brain. To see if these data could be confirmed within our previous datasets we re-interrogated data we had obtained from a proteomics analysis of degenerating versus control retina. We found that Hb beta subunits 1 and 2 had previously been detected as changed in those datasets and Hb appeared reduced in quantity in those whole retina preparations from *pcd^5j^* mice.

The production of haem in the mitochondrion has long been known, but the Hb proteins have not previously been identified within the mitochondrion. Though ‘omics’ approaches have opened the door to a new type of discovery based biology there are preconceptions and intellectual filters that may prevent biologists from examining a data point that appears ‘unlikely’or ‘contamination’. The combination of these ‘filters’ along with the torrent of data that results from a proteomics study can leave many potentially interesting ‘hits’ unexplored. Mitoprot II (http://www.bio.net/mm/comp-bio/1997-April/001331.html) prediction scores for sequences corresponding to Hba and Hbb entering the mitochondrion are 0.2 and 0.19 respectively. We decided that further experimental work needed to be done to confirm our findings. We determined to follow up on the possibility that the presence of Hb proteins within the mitochondrion may have been overlooked up until now.

Recently, it has been shown that Hba has an intracellular role in endothelial cells, regulating nitric oxide signalling (Straub et al., 2012). Previously Hb has been detected in neurons and found to be reduced in AD and PD (Ferrer et al., 2011a; IOS Press, n.d.). We wanted to know whether Hb plays a role in maintaining the mitochondrial milieu and has a supporting role in the process of oxidative phosphorylation. In order for this to be the case, we hypothesised that Hb would need to be present in close proximity to the location of the ox. phos. machinery—within the inner mitochondrial membrane and inter-membrane space. Our ultra-structural studies revealed rosette formations of immunogold particles denoting the inclusion of Hbs in the inner mitochondrial membrane areas. The rosette arrangement of gold particles appears to be characteristic for Hbs, and may indicate a tertiary structure adopted by Hb, within this organelle. It is interesting that the most well studied oxygen transporter is localised to the very region of the organelle where oxygen is required. There are many possible explanations which now need to be followed up. An obvious and attractive idea is that the Hbs maintain a sufficient level of oxygen in close approximation to the oxidative phosphorylation machinery to prevent a potential crisis due to local hypoxic events. Hb also has the potential to buffer the inter-membrane environment, through the acceptance of protons (hydrogen ions) by histidine residues. Buffering within the mitochondria could be useful in protecting the organelle against oxidative species, for example, in the control of nitric oxide mediated apoptotic events.

Clues about the normal role of Hb in the mitochondrion are likely to be gained by examining situations in which we know mitochondrial function is altered. Mitochondrial function has been examined at some depth in neurodegeneration. Our approach used a classic mouse model of neurodegeneration, *pcd^5j^*, which we have previously shown to have features of mitochondrial dysfunction (Chakrabarti et al., 2010). A primary risk factor for neurodegeneration is advanced age. There are several avenues of research into the mitochondrial basis of ageing which may converge in the study of the Hb proteins as a component of the mitochondrial mix. The discovery of mitochondrial Hb pulls together the oxidant theory; factors affecting the plasticity of the organelle's response to its environment and energy adaptations required throughout life and in disease (Peterson et al., 2012). Diseases of ageing encapsulate the major health threats of our time, including metabolic syndromes, cancer and neurodegeneration. Our comparison of ‘young’ and ‘old’ adult mouse tissues suggests that the different Hb proteins may be modulated between the mitochondrion and cytosol in a tissue specific manner.

The most common forms of age-related neurodegeneration are AD and PD, both have been connected with mitochondrial dysfunction (Yan et al., 2012). In the field of PD, the effort to elucidate the role of this organelle stemmed originally from the observation that mitochondrial toxins in mammalian models can elicit a similar pathology to the human condition (Perfeito et al., 2012). In recent times mitochondrial dynamics (including mitophagy via the PINK1—Parkin pathway), complex I deficiency and ROS production have all been implicated—tying the mitochondrion's functional status to PD onset and pathology (Chu, 2010; Santos and Cardoso, 2012). We find that there appear to be qualitative and quantitative changes in the localisation of the Hbs to mitochondria in human neurodegenerative disease. Double staining of frontal lobe (AD) and substantia nigra (PD) revealed co-localisation of mitochondrial and Hb proteins in the affected tissues. Neurons in the substantia nigra of PD brain were particularly striking. Cellular fractionation revealed an overall picture that could be interpreted in a variety of different ways. PD mitochondrial fractions show a significant loss of Hbs from the organelles into the surrounding cytoplasm. A possible explanation is the dramatic loss of affected nigral neurons in PD, which could skew the data to look as though there is a reduction in mitochondrial Hb. Further detailed studies of staged post-mortem AD and PD brains should reveal more about these processes. It is likely that Hb localisation and function are dynamic and tissue specific in nature. This is demonstrated by our findings in the *pcd^5j^* mouse. Analyses of these proteins in their intracellular context are likely to reveal more about the process of neurodegeneration.

Haemoglobin is a member of a large family of globular proteins, the globins. Globins are characterised by a series of eight alpha helical sections that together make up what is termed the globin fold. The globins are an ancient protein family and can be found in bacteria and archaea as well as eukaryotes (Moens et al., 2007). A single species can utilise several globin proteins simultaneously. Though these are well studied proteins it is important to note that they carry out diverse functions, from their well-known oxygen binding ability to nitric oxide detoxification, regulation of gene expression and terminal oxidase activity (Vinogradov et al., 2006).

There have been previous reports connecting haemoglobin and neurodegeneration (Straub et al., 2012). However, one of the most intriguing ideas to arise from this study is whether we are now able to make molecular connections that directly link the effects of hypoxia and or physical exercise (oxygenation of Hb), to dementia and neurodegeneration. Hypoxia is known to affect amyloid β (Aβ) expression and we also know that Aβ interacts with Hb in AD plaques (Chuang et al., 2012; Daulatzai, 2013). It may be that amyloid is responsible for sequestering excess Hb and reactive Fe (II) ions from degenerating neuronal mitochondria. An iron detoxification role for another protein important for neuronal health, Frataxin (FXN), has been demonstrated (Gakh et al., 2006). FXN is localised to the inner-membrane and inter-membrane space of the mitochondrion and should now be investigated in relation to the presence of the Hb proteins in close proximity. Processed Hb from dysfunctional mitochondria could be responsible for the iron-load observed in AD and PD brains (Ferrer et al., 2011b; Sian-Hülsmann et al., 2011). Weak evidence connecting anaemia and PD can now be investigated by more robust methods (Savica et al., 2009). Anaemia is a common finding in the elderly, it would be interesting to investigate a link between circulating Hb levels and mitochondrial Hb (Gaskell et al., 2008).

The discovery of Hb proteins within the functional regions of the mitochondrion has immediate and obvious implications in many areas of biology. We have begun to explore aspects of ageing and neurodegeneration. Metabolic syndromes also display altered functions of mitochondria (Hu and Liu, 2011). It is also possible that the Hb content and function of cancer cell mitochondria might be altered, maybe leading to a fuller explanation for the altered metabolism in tumour cells (Wallace, 2012). Further work to characterise the role of Hbs in the mitochondrion is likely to reveal answers to the most basic questions concerning cellular metabolism and will shed light on many clinical conundrums.

## 4 Methodology

### 4.1 Mitochondrial isolation

Crude mitochondria were extracted as described previously (Chakrabarti et al., 2010). Sub-mitochondrial fractions were prepared essentially as described previously except the primary inner membrane fraction was not further processed to the final inner membrane and final matrix fractions. The four fractions thus obtained were called the inner membrane, the matrix, the outer membrane and the inter-membrane space.

### 4.2 2dimensional (2D) gel electrophoresis

Brain tissue was obtained from 27 day old *pcd^5J^* (http://jaxmice.jax.org/strain/004518.html) mice and controls. Mitochondria were isolated from sample tissue. Mitochondrial protein lysates were used for 2-D gel electrophoresis. Samples were subject to iso-electric focussing using the ZOOM IPG (Life Technologies) system and pH 4–10 (non-linear) ZOOM IPG strips following the manufacturer's protocol. The second dimension of separation was carried out using Novex 4–12% Bis-Tris ZOOM gels at 150 V for 70 min. Gels were stained with SYPRO Ruby (Life Technologies) fluorescent dye following the manufacturer's protocol. The stained gels were scanned and visualised using a Typhoon Trio scanner (GE) at a voltage rating of 450 pmt, using the green filter at a 200 micron resolution. Gel analyses used SameSpots software (Nonlinear Dynamics, Newcastle upon Tyne, UK). Gels for mass spec analyses were silver stained. Spots were picked using a manual spot picker and submitted for tandem mass spectrometry (Susan Liddell, South Lab proteomics facility, Sutton Bonington Campus, University of Nottingham).

### 4.3 Spot processing and tryptic digestions

Protein samples were excised from gels manually using a gel spot cutting pen and the samples were processed in gel pieces using the ProteomeWorks MassPREP robotic liquid handling station (Waters, Elstree, UK). The samples were incubated three times, in 100 μl of de-stain solution (50 mM ammonium bicarbonate, 50% acetonitrile) for 10 min at room temperature. Following removal of the final aliquot, the sample was dehydrated by incubation at room temperature in 50 μl of acetonitrile for 5 min, acetonitrile was removed and the gel plugs incubated for 10 min to allow evaporation. The sample was further processed by incubation at room temperature in 50 μl of reducing solution (10 mM dithiothreitol, 100 mM ammonium bicarbonate) for 30 min and, following removal of the reducing solution, incubation at room temperature in 50 μl of alkylation solution (55 mM iodoacetamide, 100 mM ammonium bicarbonate) for 20 min. The gel slice was then washed at room temperature in 50 μl of 100 mM ammonium bicarbonate for 10 min, 50 μl of acetonitrile for 5 min and dehydrated by double room temperature washes in 50 μl of acetonitrile for 5 min and evaporation for 5 min. The microtitre plate containing the gel plugs was cooled to 6 °C for 10 min before addition of 15 μl per well of trypsin gold (Promega), diluted to 10 ng μl^− 1^ in trypsin digestion buffer (50 mM ammonium bicarbonate). The plate was incubated at 6 °C for a further 20 min to permit trypsin entry into the gel plugs with minimal autocatalysis before incubation at 40 °C for 4 h. Samples were stored at 4 °C until mass spectrometry (MS) analysis.

### 4.4 Tandem MS

Samples were analysed by LC–ESI-MSMS on a Q-TOFII mass spectrometer fitted with a lockspray nano-ESI (electrospray ionization) source (Waters Ltd.). Peptides were captured and desalted on a C18 cartridge and delivered on-line via a C18 column to the MS via a CapLC HPLC system. The mass spectrometer was operated with a capillary voltage of 3000 V in positive ion mode, using argon as the collision gas. Tandem MS data were acquired using an automated data-dependent switching between MS and MS/MS scanning based upon ion intensity, mass and charge state (data directed analysis (DDA™)). Charge state recognition was used to select doubly, triply and quadruply charged precursor peptide ions for fragmentation. One precursor mass at a time was chosen for tandem MS acquisition. Collision energy was automatically selected based on charge and mass of each precursor and varied from 15 to 55 eV.

### 4.5 Tandem MS data processing and database searching

ProteinLynxGlobalServer version 2.0 (Waters, Ltd.) was used to process the uninterpreted MS data into peak list (pkl) files which were searched against all entries in Swissprot and NCBInr databases using the web version of the MASCOT MS/MS ions search tool (http://www.matrixscience.com/). Carbamidomethylation of cysteine and oxidation of methionine was set as variable modifications. One missed cleavage by trypsin was accepted.

### 4.6 Immunoblotting

Western blotting was conducted as follows: 1 μl mitochondrial extract or 6 μl cytoplasmic extract was diluted to 10 μl in phosphate buffered saline (PBS). 5 μl 4 × DTT and 5 μl 4 × LDS sample buffer (Life Technologies) were added and samples were vortexed and boiled for 5 min. Samples were loaded onto precast 12 well NuPage 12% Bis-Tris Gels (Novex, Life Technologies) and run at 200 V for 35 min. Gels were transferred at 100 V for 60 min to 0.45 μm Nitrocellulose membrane (Novex, Life Technologies). Membranes were blocked in 3% (w/v) BSA (Bovine serum albumin) in TBS-T (Tris-Buffered Saline and 0.1% Tween-20) for 1–2 h at room temperature, then incubated at 4 °C overnight in primary antibodies (Hba ab102758 (Abcam) 1:500; Hba sc-21005 (Santa Cruz) 1:1000 or Hba sc-31332 (Santa Cruz) 1:1000; Hbb sc-22718 (Santa Cruz) 1:1000; VDAC/Porin ab15895 (Abcam) 1:2000; NDUFs3 ab110246 (Abcam) 1:1000; SMAC/Diablo ab8115 (Abcam) 1:1000; COXIV ab16056 (Abcam) 1:1000; Histone H3 ab1791 (Abcam) 1:1000; Hsp-90 ab13495 (abcam) 1:500; dilution in 3% (w/v) BSA in TBS-T). Membranes were washed 3 × 5 min at room temperature in TBS-T and incubated in peroxidase conjugated rabbit anti-goat IgG, goat anti-rabbit IgG, or rabbit anti-mouse IgG (Invitrogen), as appropriate, for 2 h at room temp, at a 1:4000 dilution in 3% BSA (w/v) in TBS-T. Membranes were further washed 3 × 5 min in TBS-T and developed using ECL Prime (GE Healthcare) for 5 min. Proteins were visualised using a Typhoon Trio Imager (Amersham Biosciences). Band densities were measured using image J. Cytoplasmic samples were normalised to hsp-90 and mitochondrial samples were normalised to VDAC/porin. Using the normalised values the ratio of mitochondrial/cytoplasmic HbA and HbB were calculated. Plotted are mean +/− SEM. Statistics were calculated using Graphpad prism (eg. one-way or two-way anova or *t*-test depending on which figure).

### 4.7 Immunohistochemistry

Brain sections were obtained from, Human Tissue Authority approved, Nottingham Health Science Biobank (Nottingham University Hospitals NHS Trust). Immunohistochemistry was performed using standard procedures. Briefly, slides were de-waxed and rehydrated, Image-iT FX signal enhancer (Invitrogen) was applied and incubated for 30 min at room temperature. Antigen retrieval was completed using 10 mM tri-sodium citrate, pH 6.0 for 10 min at a medium heat. Slides were blocked using 5% (w/v) BSA in TBS for 1 h at room temperature, then incubated with primary antibodies: HbA, sc31332 (Santa Cruz), 1:1000, or HbB, sc22718 (Santa Cruz), 1:1000, for 1 h at room temperature. Slides were washed 2 × 5 min in PBS, and then incubated with donkey anti-goat Alexa Fluor 488 (Invitrogen), 1:200, for 1 h, in the dark, at room temperature. Slides were washed 2 × 5 min in PBS, then incubated with anti-COXIV, ab16056 (Abcam), 1:1000, at 4 °C, in the dark, overnight. Slides were further washed 2 × 5 min in PBS and incubated with goat anti-rabbit Alexa Fluor 532 (Invitrogen), 1:200, for 1 h, in the dark, at room temperature. Slides were further washed 2 × 5 min in PBS and DAPI (1 μg/ml, Sigma) added for 10 min at room temperature before applying Vectashield (Vector Laboratories, Inc.) and coverslips. Slides were visualised using a DM5000B Upright microscope (Leica) and images captured with a DC350 camera (Leica).

### 4.8 Immunogold transmission electron microscopy

Fully consented human brain tissue was obtained from The Nottingham Health Science Biobank (Nottingham University Hospitals NHS Trust). Tissue was embedded in 10% agarose and 200 μm slices cut using a LEICA VT1000S vibratome. Tissue sections were blocked in 10% PBS/BSA/Tween 20 (buffer1). Sections were incubated with ante-HbA (as before-Santa Cruz) 1:25 overnight. Specimens were washed 4 × 5 min with buffer 1 before adding the secondary gold antibody GAR-20910/2 (Aurion) 1:500 at room temperature for 4 h. Samples were washed 4 × 5 min with Buffer 1 and rinsed 4 × 5 min with PBS. Samples were dehydrated and embedded in Araldite resin (TAAB Laboratories Equipment Ltd.). 110 nm slices were transferred to 200 mesh copper-grids, and contrasted with lead citrate and uranyl acetate prior to imaging.

### 4.9 Human tissues

Human brain sections and frozen brain samples were obtained from, Human Tissue Authority approved, Nottingham Health Science Biobank (Nottingham University Hospitals NHS Trust). Tissues used for western blotting were frozen at the time of post mortem (PM). Frozen material from human cortex was used for immunoblotting studies. Material used for histology and immune EM studies were fixed in PFA at the time of PM. PM delay varied from a minimum of 3 days after death to a maximum of 6 days. Diagnoses of AD and PD confirmed at PM. Age at death varied from 59 to 81 years for PD brains and 80–85 years for AD brains, non-degenerative human control brains were age matched to within five years of AD and PD samples.

### 4.10 Mouse tissues

Young (8 weeks) and old (18 months) C57BL6J frozen brain and skeletal muscle were obtained (Charles River Laboratories, Inc.) and used to prepare cellular fractions as described. *Pcd^5j^* (www.jax.org) were a kind gift from AR La Spada (UCSD, USA). Tissues were obtained after animals were humanely killed, by a trained individual, using a Home Office approved Schedule 1 method of killing. Animals were bred and housed in accordance with strict Home Office stipulated conditions. The overall programme of work (in respect to the original UK Home Office Project Licence application) is reviewed by the Animal Welfare and Ethical Review Body at the University of Nottingham and then scrutinised by the UK Home Office Inspectorate before approval by the Secretary of State. Individual study protocols link to the over arching Home Office Project Licence and are made available to the Named Animal Care and Welfare Officer, the Named Veterinary Surgeon (both are members of the AWERB), the animal care staff and the research group. The Project Licence Number for the breeding and maintenance of this genetically altered line of mice is PPL 40/3576. The mice are typically group housed and maintained within solid floor cages containing bedding and nesting material with additional environmental enrichment including chew blocks and hiding tubes. Cages are Individually Ventilated Cage Units within a barrier SPF unit to maintain bio-security. Animals are checked daily by a competent and trained animal technician. Any animal giving cause for concern such as subdued behaviour, staring coat, loss of weight or loss of condition will be humanely killed using a Home Office approved Schedule 1 method of killing.

### 4.11 Gene Ontology (GO) analyses (Alexa et al., 2006)

GO analysis is performed with the Bioconductor package topGO which compares three different methods, classic, elimination, weight. *Workflow*: 1- Using the all known universe (18314), analyze the Purkinje MUT/WT proteins and the other cerebellum MUT/WT proteins lists: a) up AND down, b) up only, c) down only. 2- For each analysis output a summary file with p-values for the three methods, output a summary file of the top 50 GO terms determined by each method so you can see how each GO term was ranked by the three methods, output another summary file of the top 50 GO terms including which proteins from each contrast were found in the various GO terms.
